# The Southern Surgical and Gynecological Association

**Published:** 1897-12

**Authors:** 


### THE SOUTHERN SURGICAL AND GYNECOLOGICAL ASSOCIATION.

This Association, which has now become one of our most important and scientific medical bodies, held its tenth annual session at the Southern Hotel, St. Louis, November 9th, 10th and 11th, and though the prevalence of yellow fever in a portion of the country tributary to this association no doubt kept some physicians away, quite enough were present to secure a successful and interesting session.

The President, Dr. George Ben Johnston, of Richmond, delivered an address upon "The Prevalence of Specialism, and who shall be Specialists." He thought it a mistake for young men to get the idea, while still at college, that they must follow a special study and neglect other important branches of medicine. He regarded that an experience of at least five years in the general medical field was necessary before a man undertakes to devote his entire attention to any specialty. "The present system has many disadvantages and permits unqualified men to foist themselves upon the public as specialists." The remedy must be accomplished, he said, through the instrumentality of the medical colleges and societies, and students should be warned of the folly of attempting excellence in one line before they had fully explored the entire field of medical science.

Dr. C. A. L. Reed, of Cincinnati, next read a paper upon "Gall-Stones in their Relation to Cancer of the Gall-Duct," reporting four cases in which the history of malignant diseases preceded the symptoms of gall-stones.

Dr. George H. Noble reported "Four Cases of Abscess of the Uterus." In these cases abscesses were found in the uterine walls, opened, curetted and cauterized. The cases showed that " it is not always necessary to extirpate the womb in suppurative inflammation of its parenchyma, and that infection of a puerperal uterus does not necessarily mean that the entire organ is hopelessly contaminated."

Dr. W. D. Haggard, of Nashville, presented a paper upon the "Disposal of the Stump in Appendicitis Operations," in which he approved Deaver's method of total excision of the appendix with closure of the hole in the head of the colon.

Dr. J. Wesley Bovee, of Washington, D. C., called attention to cases of "Tubal and Ovarian Hemorrhage Resembling Ruptured Ectopic Pregnancy." Among the hemorrhages which may be mistaken for ectopic pregnancy were those from malignant disease of the uterus, appendages or rectum, from varicose veins of the broad ligaments, and from inflammatory diseases of the ovaries and tubes.

In a paper on "Cystic Disease of the Mamma," Dr. L. M. Tiffany, of Baltimore, advocated removing the entire breast; he believed that other portions of the breast are so often involved that a recurrence of the growth is not uncommon.

Dr. J. G. Earnest, of Atlanta, reported a case of "Extra-uterine Pregnancy operated upon at the seventh month." Dr. Brokaw, of St. Louis, made some remarks upon the "X-Ray in Surgery," and exhibited a large number of instructive radiographs.

Among other interesting papers were: "Ovariotomy in the Aged," by Dr. A. W. Cartledge, of Louisville; "Pyuria," by Dr. H. A. Kelley, of Baltimore; "Operative Treatment of Enlarged Prostate," by Dr. H. H. Grant, of Louisville; "Chronic Proctitis," by Dr. F. F. Talley, of Birmingham; "Early Diagnosis and Treatment of Cancer of the Uterus," by Dr. W. H. Myers, of Fort Wayne. Dr. Tuholske, of St. Louis; Dr. Hall, of Cincinnati; Dr. Engelmann, of Boston, and Dr. Davis, of Birmingham, also read papers.

The next meeting of the association will be held in Memphis the second Tuesday in November, 1898.

The following officers were elected for the ensuing year: President, Dr. Richard Douglas, of Nashville, Tenn.; Vice-Presidents, Dr. H. H. Mudd, of St. Louis, and Dr. J. A. Goggans, of Alexander City, Ala.; Secretary, Dr. W. E. B. Davis, of Birmingham, Ala.; Treasurer, Dr. A. M. Cartledge, of Louisville, Ky.; Council, Dr. George Ben Johnson, of Richmond, Va.; Dr. Louis M. Tiffany, of Baltimore, Md.; Dr. Lewis E. McMurtry, of Louisville, Ky.; Dr. Geo. J. Engelmann, of Boston, Mass., and Dr. Ernest S. Lewis, of New Orleans, La.

---

A DAMAGE suit involving a novel question has just been tried in Chicago. A woman, within four days of her confinement, entered a lying-in hospital, and in going up the elevator some accident happened to the latter, by which her unborn child was permanently injured. This child afterward became the plaintiff in the suit. "The question was whether a child after it is born has a right of action for injuries sustained by it *en ventre sa mère;* in other words, whether a child unborn is a person in being, so as to be entitled after its birth to maintain such an action." Judge Chetlain rendered a decision in the affirmative, holding that the plaintiff had good cause for action.

---

FOLLOWING are the officers elect of the Mississippi Valley Medical Association: President, Dr. John Y. Brown, of St. Louis; Vice-Presidents, Dr. A. J. Ochsner, of Chicago, Dr. A. P. Buchanan, of Fort Wayne; Secretary, Dr. H. E. Tuley, of Louisville; Treasurer, Dr. C. A. Wheaton, of St. Paul. Next place of meeting, Nashville, second Tuesday in November, 1898.

